# Programs to encourage working as a general practitioner in rural areas: why do medical students not want to participate? A cross-sectional study

**DOI:** 10.1186/s12909-022-03688-x

**Published:** 2022-08-17

**Authors:** Nikolaos Sapoutzis, Antonius Schneider, Tom Brandhuber, Pascal O. Berberat, Marjo Wijnen-Meijer

**Affiliations:** 1 Technical University of Munich, School of Medicine, TUM Medical Education Center, Ismaninger Straße 22, 81675 Munich, Germany; 2 Public Health Department Hochtaunuskreis, Bad Homburg vor der Höhe, Germany; 3 Technical University of Munich, School of Medicine, Institute of Family Medicine and Health Services Research, Munich, Germany

**Keywords:** Family medicine, Rural areas, Specialisation, Motivation, Curriculum development

## Abstract

**Background:**

In many countries, not enough students are interested to work as general practitioners in rural areas. To solve this problem, several, sometimes partly extracurricular, programs have been developed. Most of these programs are based on continuity, which means that students stay in a rural region for an extended period of time, by completing clerkships. Although the effects of these programs are positive, it is often difficult to motivate students to participate. The purpose of the present study is to get insight into the reasons why students choose not to participate in these programs.

**Methods:**

We carried out a questionnaire study among medical students in the clinical phase of the Technical University of Munich in Germany. First, we asked the students whether they actively informed themselves about the program which aims to reduce the shortage of general practitioners in rural areas in Bavaria. Furthermore, the questionnaire focused on the reasons for not participating in this program.

**Results:**

Based on the answers of 442 students from study years 3–6, the most frequently chosen reason for not participating in the program is “identification with another discipline” with 61.0%, directly followed by “not willing to commit long-term” (56.1%). In third place is “personal connections to another region” with 30.5%. In the open comments, we find the same reasons: many students do not want to commit to a certain direction too early. In addition, students indicate that the number of regions where this program is offered is too limited for them.

**Conclusions:**

Offering programs to prepare and motivate students for work as general practitioners in rural areas can contribute to increasing the pool of future general practitioners. To encourage students to participate in such a program, it is important to consider the motives of students. Many students who might be interested in general practice do not choose to take part in such a program because they do not want to commit to a particular specialty or region at an early stage. It is important to take these insights into account when designing and implementing these programs.

## Introduction

Choosing a specialisation is probably the most important decision medical students have to make. This choice is not only important for the students themselves, but also for society. The aspirations of medical students do not always match the needs of society. For example, in many countries too few students are interested in a specialisation in family medicine, psychiatry or Gynecology, which will create a future shortage of physicians in these specialties [1–6] . Factors that are taken into account are the research possibilities [3, 7], the perceived status of the discipline [8, 9] and the patient population [7, 10]. This problem is not new - in the 1960s, too few medical students were interested in a career as a general practitioner as well [11]. Another problem in many countries is that not enough students are motivated to work in rural areas [2, 12–14]. Research shows that the reasons for this are both personal and professional. Factors of a personal nature are fear of social isolation and limited work opportunities for their partners [7, 15–17]. Regarding the work conditions, these include the fear of a higher level of responsibility, less support from a team and fewer opportunities for networking [7].

To encourage students already during medical school to work in rural areas, many countries have developed specific programs, often with a focus on family medicine [16–21]. Most of these programs are based on continuity, which means that students stay in a rural region for an extended period of time, by completing clerkships [14, 16, 19, 22, 23]. In this way, students experience what it is like to work and live in such an environment [13, 24]. In addition, they can become part of the community life and build connections with physicians and patients [17, 23, 25].

Much research has been done on the effects of such programs. Students who participated recognised that the rural clerkships expedited both their personal and professional growth [13, 25]. Conducting clerkships in rural settings prepared students for working and living in these areas [13, 24] and positively influenced graduates´ desire to practice rural medicine or family medicine [17, 25]. They appreciate being part of a team, fulfilling more of the role of a physician and being able to follow patients over a longer period of time [17]. The connections with community members outside the clinical context made students more empathetic and responsive to the needs of rural patients [17, 25]. In general, according to stakeholders, these programs deliver competent physicians [16]. After participating in rural programs with a focus on family medicine, students were significantly more active in the tasks of clinical assessment, ordering and interpreting results, and preparation of a management plan [26]. In these approaches, it is a major benefit for students to see and learn about the continuity of primary care and the whole life cycle of health and disease [27].

An important finding is that more graduates of well-organised rural programs choose to practice in underserved or rural areas [18, 28]. The quality and the duration of the rural immersion experience are important for decision-making about working in a rural area after graduation [18]. A review comparing short and long rural experiences concluded that there was an overall positive correlation between length of clerkships and interest in working in a rural area [19]. Longer placements last around twelve-months to three-years, while shorter experiences lasting 1 month are not having a significant impact on the decision to work in rural areas after graduation [19, 22]. In addition, rural rotation had a positive effect on future practice locations across all disciplines including family medicine, non-surgical specialties, and general surgery [19]. The strongest association was for a combination of clerkships in regional hospitals and rural general practice [22]. In addition to positive experiences with the clerkships themselves, participation in community life is an important prerequisite for the decision to work as a physician in rural regions [23]. Even students who have had negative experiences during the clerkships, felt lonely or have no interest in working in rural regions still find such a program a valuable addition to the regular curriculum. It gives the students the opportunity to develop personal and social competences, which are also useful in other work environments [25].

In addition to the effects of such programs, research has also been done to determine which students are interested in working in rural areas. Mostly students with rural backgrounds themselves, or with a partner of rural origin are motivated to work in rural areas after graduation [2, 15, 18, 19, 22, 29–31]. Financial support can also contribute positively to this decision [31]. Another interesting factor influencing the decision to work in rural practice is the graduate entry-level. Students who have already obtained another tertiary degree before medical school, are more likely to work in rural practice than students with school leaver entry [32]. Especially graduates from a humanities, commerce, business or law background are significantly more likely to choose a rural area to work after graduation from medical school [32]. Other personal factors supporting the decision to work as a general practitioner in rural areas are experience with working in developing countries or other social activities, doing volunteer work prior to medical school, a desire for a broad spectrum of practice, a holistic work approach, lower social class background, having young children and a stronger interest in social problems [2, 15, 33].

In summary, based on the literature, it can be said that especially students with a rural background, widening experiences prior to medical school and an interest in pursuing a broader specialisation are potentially interested in working in rural areas after graduation [2, 15, 18, 22, 31–33]. It is also known that doing clerkships in a rural setting for an extended period of time often tends to have a positive effect on the decision to work there [18, 19, 22]. Although the literature shows that students with a rural background are more likely to work as physicians in rural areas, this is not a certainty and there are several other factors that play a role, such as opportunities for the partner [29, 30]. Therefore, simply admitting more students with a rural background to medical school will not solve the problem. In order to reach a large enough group of potential future country physicians, it is important to know the reasons why students do not want to participate in programs that aims to encourage working in rural regions.

The aim of the study described in this paper therefore was to gain insight into students’ reasons for not participating in educational rural programs during medical school. The main research question is:

What are the reasons of medical students in the clinical phase for not participating in a program that aims to encourage working in rural regions?

Subordinate questions of the study are:

- Do the reasons mentioned differ between students of different gender?
- Do the reasons mentioned differ between students of different ages or backgrounds?

## Methods

### Context

In the summer of 2018 the Institute of Family Medicine and Health Services Research of the Technical University of Munich, had launched a new program (BeLA – “Beste Landpartie Allgemeinmedizin”) for medical students, which aims to reduce the shortage of general practitioners in rural areas in Bavaria [1]. The participating students are offered an extracurricular program, which includes education in small groups on topics relevant to family medicine and mentoring by an experienced general practitioner. The students are linked to one of the three rural regions participating in the project, where they complete a substantial part of their regular clerkships. Through the multiple-year extracurricular program, mentoring and the fact that they do several clerkships in one of the regions, an identification with family medicine in general and with the respective region in particular can be established. In order to improve clinical education in the concerned regions, didactic training for the supervising physicians takes place regularly. The participating students are also financially supported, by means of a monthly allowance. Attached to this is the obligation to follow residency training in family medicine for a certain period after graduation (depending on the length of the period for which they received the financial support). If they do not, they must repay the grant received. The program has a positive effect: students who participate are more likely than average to opt for residency in family medicine after graduation, often in the region where they did their clerkships or for another residency program in this rural region [1]. However, it turns out to be difficult to motivate students to participate in the BeLA program. This mainly concerns the 4-year program that runs during the entire clinical phase. There is also an option for students to participate in the program only in the last year of medical school, which more students are willing to do. Students are informed about the project through flyers, posters, information sessions and intranet.

### Sample

The present study was part of a larger cross-sectional study regarding motivation for an identification with family medicine. In December 2020 questionnaires were sent electronically to all 1643 students in the clinical phase (year 3–6) of the medical school of the Technical University of Munich. The invitation to participate was sent by email that contained an explanation of the purposes of the study and a link to the questionnaire. Two weeks after the initial mailing, a reminder was sent.

### Questionnaire

To gather information about the reasons why students do not want to participate in the BeLA-program, we designed and administered a questionnaire for medical students. The first part of the questionnaire consisted of questions on the background of the respondents: age, gender, whether they have already completed a higher education program and whether they have children. The students were asked if they participate in the BeLA program (yes/no). The students who answered “no” to this question were then asked the following two questions:

1. Did you actively inform yourself about the BeLA program, by looking for or requesting information: yes/no.
2. What are the reasons for not participating in the BeLA program? Eight response options were given - 7 specified reasons and the option “other” (see Table 2). These reasons are based on literature describing why (future) doctors do not want to work in rural areas:

Identification with another discipline [12]; personal connections to another region [7, 15, 16]; unwilling to work in the countryside [31]; parents/partner are/is settled in another region [7, 15]; financial support is insufficient [15, 31].

This list is supplemented by reasons given by students in communication with the project staff: “not willing to commit long-term” and “planned to take over the practice from parents/acquaintances in another region”.

The students could choose multiple reasons. If the students selected the option “other”, they could clarify this in a text field.

### Data analysis

Statistical analysis was performed with IBM SPSS Statistics 27 software (SPSS Incorporated, Chicago, USA). We used descriptive statistics to describe demographic data. Chi-square tests were used to calculate differences between groups with regard to the reasons chosen. A *p*-value of < 0.05 was considered statistically significant.

The procedure for analysing the comments under “other” was as follows:

Two researchers independently ranked the comments and assigned themes - without a previously established coding scheme. A third researcher compared them. Issues for discussion were then discussed in a meeting with the three researchers, after which the themes were finally determined.

### Ethical approval

Approval for the study was obtained from the Ethics Committee of the Faculty of Medicine of the Technical University of Munich (approval no. 627/19 S). The survey was anonymous and voluntary, and all participants gave consent in accordance with the Declaration of Helsinki. All students received information on the nature, purpose and procedure of the survey and their right to withhold or revoke their consent at any time.

## Results

### Respondents

Of the 1643 students, 449 were returned giving a response rate of 27.3%. Of the respondents, 73.3% were female. Women are slightly over-represented among the respondents compared to the total student population (68%) of the medical school of the Technical University of Munich. The average age of the respondents, 24.6 years, is in line with the average age of the total student population. The distribution across the study years varies between 18.9 and 26.6%, with year 4 (second clinical year) slightly overrepresented (see Table 1). Of the respondents, 4.5% had one or more children and 21.2% had completed another degree before medical school. The latter is considerably higher than the total student population. Although the exact numbers are not known, it is estimated to be around 5%.

**Table 1:** Distribution of respondents across years of study

Year of study	% of the respondents
3	21.3
4	26.6
5	24.6
6	18.9
Unknown	8.6

### General interest in the BeLA program

Of the respondents, 7 students participate in the BeLA program. These students have been excluded from the further analysis of the results. Of the other 442 students, 49.8% indicated they have actively informed themselves about the BeLA Program, by searching the internet, going to information sessions and/or contacting staff members involved in this project.

### Reasons for not participating in the BeLA program

We asked the students about their reasons for not participating in the BeLA program. They were invited to choose all applicable answers from a list of 7 possible reasons (see Tables 2 and 3). The most frequently chosen reason is “identification with another discipline” with 55.7%, directly followed by “not willing to commit long-term” (56.1%). In third place is “personal connections to another region” with 30.5%.

**Table 2:** Reasons for not participating in the BeLA-Project (multiple choice possible) – Total and by gender

Reason	Total %	Male %	Female %
Identification with another discipline	55.7	50.0	57.7
Not willing to commit long-term	51.1	43.2	54.0
Personal connections to another region	27.8	23.7	29.3
Unwilling to work in the countryside^a^	21.9	24.6	21.0
Parents/partner are/is settled in another region^b^	21.0	11.9	24.4
Financial support is insufficient^c^	5.4	11	3.4
Planned to take over the practice from parents/acquaintances in another region.	2.7	2.5	2.8
Other	12.7	10.0	13.4

^a^Significantly less students who have already completed another study before entering medical school

^b^Significantly more females

^c^Significantly more males

**Table 3:** Reasons for not participating in the BeLA-Project (multiple choice possible) – distribution over the years of study

Reason	3rd Study year (***n*** = 95)	4th study year (***n*** = 119)	5th study year (***n*** = 110)	6th study year (***n*** = 82)
Identification with other discipline	63.2%	52.1%	74.5%	48.8%
Not willing to commit long-term	66.3%	53.8%	48.2%	35.4%
Personal connections to another region	27.4%	27.7%	28.2%	26.8%
No willingness to work in the countryside	27.4%	21.8%	21.8%	17.1%
Parents/partner are/is settled in another region	15.8%	27.7%	23.6%	17.1%
Financial support is insufficient	6.3%	5.0%	4.5%	4.9%
Planned to take over the practice from parents/acquaintances in another region.	2.1%	5.0%	1.8%	0%

After analysing the multiple answers, we saw that some answers were given in combination. Most of the students who chose “financial support is insufficient”, also chose “not willing to commit long-term”. There was often a combination of the answers “Parents/partner are/is settled in another region” and “Planned to take over the practice from parents/acquaintances in another region”. Especially the students who chose “identification with another discipline” also chose “financial support is insufficient” and “unwilling to work in the countryside”.

There are no significant differences in the reasons given depending on the year of study (Table 3). If we look at the gender of the respondents, there are some differences (see Table 2). Significantly more females mention their parents or partner being settled in another region as a reason for not participating in the program (χ^2^ = 0.004, *P* < 0.05). More males state insufficient financial support as a reason for not participating. (χ^2^ = 0.002, *P* < 0.05). Another significant difference can be seen regarding the reason “unwilling to work in the countryside”. This is mentioned significantly less as a reason by the students who have already completed another study before entering medical school (χ^2^ = 0.005, *P* < 0.05).

### Other reasons

We asked the respondents who answered “otherwise” (12.7%) to comment on this. Some students chose “otherwise” as its own option, others have used it to specify the options selected. The answers can be divided into 4 groups.

The most frequently mentioned answers (38.5%) can be put down to the fact that the students do not want to commit themselves too early.

“Afraid to commit now even though it would be appealing to me.”

“The program is too much of a commitment to rural general medicine for me. I still want to keep as much as possible open to me and would rather just have a quick look.”

“I think it’s a great project but I want to leave open which specialty I want to pursue later on”.

“I don’t want to commit to a discipline yet.”

Although a few of these students also selected the option “Not willing to commit long-term”, the focus is slightly different. “Not willing to commit long-term” is about committing to a discipline and/or a region for a longer period of time. While the open comments in this category are more about the *moment* they have to make the choice.

The reason that the students do not want to work as residents in the specific regions is also often mentioned (23.1% of the comments). Examples of comments are:

“Other/more regions for [vocational training / residency program] would be interesting, which would allow compatibility with family and partner.”

“Really a pity that the project is so locally bound and you cannot do it everywhere!”

“Find it personally very interesting. A larger choice of regions would be nice.”

“Regions are very limited”.

Some students indicate that they have too little knowledge about the project (20.5% of the comments):

“I don't know what exactly the BeLa project involves, not informed enough.”

“Don't know exactly what the BeLA project is about...”

Finally, a number of students (11.5% of the comments) indicate that they are already receiving another grant, for example:

“The Bavarian government offers the same financial support for fewer commitments.”

“I have another scholarship”.

Some of these students also chose the option “Financial support is insufficient”.

As with the given reasons, we find no distinction between study years with regard to “other reasons”. Also with regard to other student characteristics, we found no differences between subgroups.

## Discussion

This study was designed to investigate the reasons why students do not want to participate in programs that prepare them to work as general practitioners in rural areas. Besides the fact that some of the students have already more or less made a choice for another discipline, we mainly see that students do not want to commit themselves to family medicine and/or a specific region at an early stage for a long period of time.

This is in line with the many studies done on the choice of specialization among medical students. Several studies have shown that for most medical students their preference for specialisation changes during medical school and often also in the period after graduation [34–37] and that females seem to make the final decision later than males [38]. A survey of German medical graduates shows that 77% of students make the decision about their choice of specialisation in the final year of medical school [39]. Querido et al. investigated the stability of specialisation preference in a longitudinal study. The results of this study show that a minority of the students stayed with the same specialisation preference during the research period of 3.5 years [40]. Singh and Alberti (2021) have examined why students change their preferences. Important reasons are the students’ experiences with different disciplines during medical school and their own assessment of whether they fit in [41]. Other studies also show that experiences during clerkships are an important factor in the final choice of specialisation [5, 42, 43]. Maybe this approach can also convince students to take part in a project such as the BeLA program mentioned in the current study. Only very few students indicate that they do not know (enough) about the BeLA program and our research shows that almost half of the students have informed themselves about this program, so there is evidence of interest.

Another important point is that the students do not want to make a long-term commitment, for example by having to pay back money if they do choose another discipline. A possible solution could be that students can also participate in the program without receiving funding. This would bring them into contact with the field and hopefully encourage them to work in it later, without feeling the pressure to make an early career decision. Financial support does not seem to be the main motivation for students anyway. Another possible approach is to limit the rural track to, for example, the last 1.5–2 years of medical school. Then the students have to make the decision to participate in the program later and the period is shorter, which possibly lowers the threshold. We know that getting positive experiences with rural family medicine can motivate students to work in that field after graduation. Almost all observational studies with comparison groups concluded that medical schools with a focus on rural health care have increasingly higher numbers of family physicians working in rural areas than medical schools of other types or non-primary care tracks [2].

Many students report that they are interested in the project and in family medicine, but do not want to live and work in the regions involved. This has several causes: some students prefer to stay in the city and some do not want to commit to working in a rural area. Most students who mention this as a reason, find the number of regions involved in the project too few and too limited. Medical schools that want to offer such a program should therefore offer as many regions as possible with hospitals and general practices where the students can do their clerkships.

Choosing a specific medical discipline is not only influenced by the curriculum and experiences at medical school, but also by the characteristics of the students [44]. A relevant finding of our research project is that students who have previously completed another degree mention not wanting to work in rural areas as a reason for not participating less often. This is in line with a study by Playford et al., who describe that graduates with prior tertiary education are more likely to work in rural areas after graduation, in comparison with students who enter medical school directly after finishing high school [32]. Although the cause of this difference is not clear, and it would be useful to investigate further, selecting more students with such backgrounds for medical school, might possibly increase the number of potential physicians in rural areas. It is striking that the group with a previous degree is overrepresented among our respondents, which may also indicate an interest in the topic.

When interpreting the results, the limitations of this study must be taken into account. A possible limitation of our study is that we asked the students about the reasons for not participating in retrospect. A possible follow-up study could be to ask students, immediately after they have received information about the project, which aspects could contribute to increasing the motivation for participating. Another limitation is the fact that the questionnaire uses fixed answers, which are largely based on the literature. This may cause a loss of themes. Although this effect seems to be limited, because the students had the option to mention other reasons, it might be interesting to choose a more open-ended qualitative approach in a subsequent study. Other factors that may play a role in the choice of specialisation may also be incorporated, such as the rural background of the students and their partners, personal network and career options.

Another limitation is the modest response rate (just over 27%). Although the distribution of backgrounds, characteristics, age and gender reflect the total population, a possible bias that students interested in family medicine may be more inclined to participate in the study cannot be ruled out. It therefore seems possible that the mentioned concerns are even more pronounced among the non-responders. As our findings are based on data from one medical school in Germany, the results cannot automatically be generalised to other populations. But since the structure of medical education in Germany is the most common model for medical education worldwide [45], there are no reasons to assume that the study population is very different. However, a similar study in other countries is desirable. On the other side, since a total of over 400 students participated, spread across all years of the clinical phase of the medical school, the results still provide valuable information, also for medical schools in other countries facing the same problems. Although other studies show that programs like the BeLA program have positive effects [1, 18, 28], the impact of such programs can be increased if more students participate.

## Conclusion

Offering programs to prepare and motivate students for work as general practitioners in rural areas can contribute to increasing the pool of future general practitioners. But in order to stimulate students to participate in such a program, it is important to take into account the motives of students. As the cohort of program participants is still rather small, we focused this study explicitly on the motives NOT to participate.

The results of this research can be used to improve the conditions for such programs so that they do not hinder possibly motivated students to participate. One of these conditions is that the students do not experience any obligation, for example by having to pay back fees if they realise at a later stage that another discipline suits them better. A second important condition is to offer the program in several regions, so that students have a larger choice of where they want to do their clerkships and their residency afterwards.

## Data Availability

The datasets generated and/or analysed during the current study are not publicly available due to data protection guidelines of the institution but are available from the corresponding author on reasonable request.
